# Anemia in children with JIA: is it really driven by hepcidin level, or by a set of factors of a chronic disease

**DOI:** 10.1186/1546-0096-12-S1-P187

**Published:** 2014-09-17

**Authors:** Andrey Egorov, Vyacheslav Chasnyk, Mikhail Kostik, Ludmila Snegireva, Olga Kalashnikova, Margarita Dubko, Vera Masalova, Tatyana Likhacheva, Elena Fedorova

**Affiliations:** 1 State Pediatric Medical University, Saint Petersburg, Russian Federation

## Introduction

Hepcidin - 25-amino acid peptide - is known to be a key regulator of systemic iron metabolism [Ganz T., Nemeth E., 2012]. Hepcidin acts indirectly through ferroportin, which is both a receptor for hepcidin and the only known exporter of iron in the human body [De Falco L. et. al., 2013]. Hyperproduction of hepcidin due to the influence of pro-inflammatory cytokines, especially IL-6, triggers to transport of iron from circulation to the storage, consequently, limiting iron accessibility for erythropoiesis [Weiss G., Goodnought T., 2005]. Thus, overproduction of hepcidin seems to be the leading mechanism of development of anemia in children with Juvenile Idiopathic Arthritis (JIA). A set of negative factors of a chronic disease, particularly disbalance of vitamins, proteins, amino acids and minerals, which are also the known causes, of anemia can weaken control of iron metabolism by hepcidin.

## Objectives

To determine how strong are the correlations of serum hepcidin level with clinical and laboratory characteristics of arthritis and iron metabolism in children with JIA.

## Methods

Ten children (8 girls and 2 boys, average age 10.2 ± 4.5 years) with 2-5 year long course of the polyarticular JIA have been enrolled in the study. Patients underwent a complete clinical and laboratory examination before treatment and at weeks 4 and 16 of the study. Clinical examination included the evaluation of articular syndrome, assessment of disease activity and efficacy of the treatment using the ACRpedi-scale. The set of laboratory data included the complete blood count with erythrocyte indices: mean corpuscular volume (MCV), mean corpuscular hemoglobin (MCH) and mean corpuscular hemoglobin concentration (MCHC). Erythrocyte sedimentation rate (ESR) and C-reactive protein (CRP) were used as markers of inflammatory activity. Serum levels of ferritin, iron, total iron binding capacity (TIBC), transferrin saturation (TSAT), soluble transferrin receptor (sTfR) and hepcidin were used to assess the iron metabolism. Linear regression and Spearman’s rank coefficient were used to describe correlations.

## Results

Serum concentration of hepcidin correlated with the inflammatory activity indices. We revealed correlation (p<0.02) of serum hepcidin concentration with the number of swollen joints (r_s_=+0.60), the number of painful joints (r_s_=+0.71), with ESR (r_s_=+0.63), with the level of CRP (r_s_=+0.87), with leukocyte (r_s_=+0.89), platelet (r_s_=+0.89) and neutrophil blood cell counts (r_s_=+0.79). Rather strong correlation of serum levels of hepcidin and ferritin (r_s_=+0.90), sTfR (r_s_=+0.51), iron (r_s_= - 0.49) and TIBC (r_s_= - 0.50) as well as with MCV (r_s_= - 0.63) and with MCH (r_s_= - 0.62) was also revealed. MCH was found to be the most sensitive to the level of serum hepcidin parameter, much more sensitive than total hemoglobin concentration.

## Conclusion

The study revealed strong correlation of the serum hepcidin level with both clinical and laboratory indicators of the inflammatory activity of arthritis, as well as with indicators of the iron metabolism. Our results support the idea that anemia in JIA-children is almost exclusively controlled by hepcidin and is caused by disturbance of iron metabolism with the development of iron-restricted erythropoiesis.

## Disclosure of interest

None declared.

